# Comorbid Insomnia and Sleep‐Disordered Breathing in Painful Temporomandibular Disorders: Association With Pain Intensity

**DOI:** 10.1111/joor.70185

**Published:** 2026-03-07

**Authors:** Alberto Herrero Babiloni, Patrícia Monteiro, Antonio Sergio Guimarães, Miguel Meira e Cruz

**Affiliations:** ^1^ Center for Advanced Research in Sleep Medicine, CIUSSS du Nord de l'île‐de‐Montréal, Hôpital du Sacré‐Coeur de Montréal Montréal Quebec Canada; ^2^ Department of Stomatology Centre Hospitalier de L'université de Montréal (CHUM) Montréal Quebec Canada; ^3^ Department of Psychology University of Montreal Montréal Quebec Canada; ^4^ Faculdade São Leopoldo Mandic Campinas Brazil; ^5^ Sleep Unit, Cardiovascular Center of University of Lisbon (CCUL@RISE), Lisbon School of Medicine Lisbon Portugal; ^6^ Centro Europeu do Sono Lisbon Portugal

**Keywords:** autonomic function, chronic pain, circadian patterns, hypoxemia, oxygen desaturation, pulse oximetry, sleep quality

## Abstract

**Purpose:**

Sleep disturbances are common in temporomandibular disorders (TMD), yet insomnia‐related complaints are often treated as homogeneous despite frequent co‐occurrence with sleep‐disordered breathing. This study examined whether sleep–pain relationships differ between insomnia alone and comorbid insomnia and sleep‐disordered breathing (COMISA) in patients with chronic TMD‐related orofacial pain.

**Methods:**

In this cross‐sectional study, 50 adults with chronic TMD pain were classified as having insomnia alone (*n* = 20) or COMISA (*n* = 30) using validated questionnaires and overnight pulse oximetry. Pain intensity (VAS), pain frequency (days/week), circadian pain patterns, subjective sleep quality (PSQI) and objective nocturnal respiratory and autonomic parameters were assessed. Group comparisons and within‐group linear regression analyses were performed.

**Results:**

Pain intensity did not differ between groups (7.45 ± 1.67 vs. 7.33 ± 1.90; *p* = 0.95). Patients with COMISA reported more frequent pain (3.37 ± 1.35 vs. 2.55 ± 1.19 days/week; *p* = 0.04) and more frequent morning pain (93.3% vs. 50.0%; *p* < 0.001). Subjective sleep quality was associated with pain intensity in COMISA (*B* = 0.200, *r* = 0.417, *p* = 0.022) but not in insomnia alone (*p* = 0.353). In COMISA, lower minimum nocturnal oxygen saturation (*p* = 0.043) and maximum nocturnal heart rate (*p* = 0.021) were associated with pain intensity; no such associations were observed in insomnia alone.

**Conclusions:**

Sleep–pain relationships in chronic TMD vary by sleep phenotype. These findings support the need for further investigation of COMISA in relation to pain, particularly in orofacial pain and TMD populations.

## Graphical abstract

In 50 adults with chronic TMD pain, insomnia alone and COMISA showed similar pain intensity and subjective sleep complaints. However, COMISA was characterised by greater pain frequency, predominant morning pain and marked nocturnal hypoxemia. Sleep quality and oxygen saturation were associated with pain intensity only in COMISA, indicating that sleep–pain relationships in TMD vary by sleep phenotype. COMISA, Comorbid Insomnia and Sleep Apnea; HR: Heart Rate ODI, Oxygen Desaturation Index; TMD, Temporomandibular Disorders; SpO_2_ , Peripheral Oxygen Saturation.

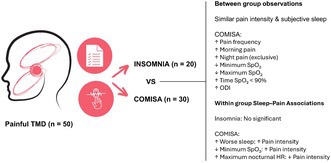

## 1 Introduction

Temporomandibular disorders (TMDs) are among the most prevalent chronic orofacial pain conditions and constitute a major source of disability, functional limitation and reduced quality of life [1, 2]. TMD‐related pain is frequently persistent, fluctuates over time and often co‐occurs with a broad range of somatic and psychosocial symptoms [3]. Among these, sleep disturbances are particularly common and clinically relevant, as sleep and pain interact through bidirectional and mutually reinforcing neurobiological, behavioural and physiological pathways [4].

Frequent sleep disturbances reported by TMD patients are insomnia symptoms, which have been widely associated with greater pain burden, increased pain‐related distress and poorer functional outcomes [4, 5, 6]. Insomnia is increasingly recognised as a modifiable factor in the management of chronic pain conditions, including TMD [7, 8]. However, effect sizes and the specific pain dimensions affected by insomnia (e.g., pain intensity versus pain persistence) vary substantially across studies, suggesting important underlying heterogeneity [9]. A significant limitation of much of the sleep–pain literature is the implicit assumption that insomnia‐related complaints represent a relatively homogeneous form of sleep disturbance. In reality, individuals reporting insomnia symptoms may differ markedly in underlying sleep physiology, sleep duration, arousal burden and co‐occurring sleep disorders.

In particular, a substantial proportion of individuals with insomnia symptoms also exhibit sleep‐disordered breathing, giving rise to another condition known as comorbid insomnia and sleep apnea (COMISA) [10]. COMISA is characterised by the coexistence of insomnia complaints and sleep‐disordered breathing–related physiological disturbances, including recurrent nocturnal hypoxemia, autonomic activation and sleep fragmentation [11]. COMISA is prevalent and has been associated with worse clinical outcomes than either insomnia or sleep apnea alone [12], including greater symptom burden, reduced quality of life, prolonged awakenings, or poorer treatment response [13, 14, 15, 16, 17]. It has been reported that these patients may have an increased risk of suicidal ideation or self‐harm [18]. Moreover, COMISA has been linked with the presence of other sleep disorders, such as sleep bruxism, although sleep bruxism does not appear to occur in patients with COMISA more frequently than in patients with OSA or those without any sleep disorders [19]. Despite this, the relevance of the COMISA to chronic pain and especially chronic orofacial pain—and TMD in particular—remains poorly understood, even though its possible presence in trigeminally mediated pain conditions such as headache has been suggested [20]. Furthermore, most prior studies examining sleep and pain rely predominantly on subjective sleep measures and do not distinguish between insomnia occurring in isolation and insomnia accompanied by physiologic sleep disruption [21]. As a result, it remains unclear whether sleep–pain associations observed in TMD reflect insomnia severity per se, or whether they are contingent upon the presence of objective respiratory‐related sleep disturbances, such as recurrent nocturnal oxygen desaturation, cumulative hypoxic burden and associated autonomic activation.

The present study aimed to examine sleep–pain relationships in a cohort of individuals with chronic TMD‐related orofacial pain by explicitly distinguishing between insomnia alone and COMISA Specifically, we sought to: (1) characterised and compare demographic characteristics, pain features, subjective sleep measures and objective nocturnal respiratory parameters between insomnia and COMISA groups; (2) determine whether subjective sleep quality are differentially associated with pain intensity across these groups; and (3) evaluate in an exploratory manner whether objective indices of nocturnal hypoxemia and autonomic activity are independently associated with pain intensity and other pain parameters. We hypothesised that subjective sleep complaints would be broadly comparable between groups, but that COMISA would be distinguished by marked physiological sleep disruption and that sleep–pain intensity associations would emerge in both groups but more strongly in COMISA group.

## 2 Methods

### 2.1 Study Design and Participants

This cross‐sectional observational study was conducted in adults with chronic temporomandibular‐related orofacial pain (TMDCOP). Participants were consecutively recruited from a specialised Orofacial Pain Clinic in São Paulo, Brazil (*São Leopoldo Mandic University*). All participants provided written informed consent prior to enrollment, and the study protocol was approved by the Ethics Committee of the abovementioned institution and conducted in accordance with the Declaration of Helsinki.

Chronic orofacial pain was defined as pain persisting for more than three months. All participants underwent standardised clinical assessment of pain characteristics, sleep symptoms and nocturnal respiratory physiology. Temporomandibular disorder diagnoses were established through clinical examination in accordance with Diagnostic Criteria for Temporomandibular Disorders (DC/TMD) [2].

Exclusion criteria included acute dental pain, current treatment with continuous positive airway pressure (CPAP), neurological disorders, major psychiatric conditions, or use of medications known to substantially affect sleep or respiration (e.g., benzodiazepines, Z‐drugs, opioids, etc.). All participants provided written informed consent, and the study was conducted in accordance with institutional ethical guidelines.

### 2.2 Pain Assessment

#### 2.2.1 Pain Intensity

Pain intensity was assessed using a Visual Analog Scale (VAS) ranging from 0 to 10, where 0 indicated ‘no pain’ and 10 indicated ‘the worst pain imaginable’. Participants were instructed to rate their average pain intensity over the preceding week, considering all painful episodes related to their orofacial condition.

#### 2.2.2 Pain Frequency

Pain frequency was assessed as the number of days per week during which participants experienced orofacial pain, with possible values ranging from 0 to 7 days per week. Participants were instructed to consider typical pain occurrence over the preceding weeks rather than isolated pain flares.

#### 2.2.3 Pain Distribution

Pain distribution was assessed through self‐report complemented by clinical examination, documenting the presence or absence of pain in predefined anatomical regions. Muscular pain categories as per DC/TMD included: Headache attributed to TMD, Myalgia and Myofascial Pain, if spreading or referral was present [2]. Specific anatomical sites assessed included the temporalis muscle, masseter muscle, temporomandibular joint (TMJ), cervical musculature, sternocleidomastoid muscle and occipital region. Each site was coded dichotomously (present/absent) based on patient report and clinical findings.

#### 2.2.4 Pain Circadian Distribution

Circadian distribution of pain was assessed by asking participants to identify the time of day during which pain was typically most prominent, categorised as morning, vespertine (afternoon/evening), nocturnal (night), or mixed (no clear time predominance).

### 2.3 Subjective Sleep Assessment

Subjective sleep characteristics were assessed using validated self‐report questionnaires widely employed in sleep medicine and pain research.

#### 2.3.1 Sleep Quality

Subjective sleep quality was assessed using the Pittsburgh Sleep Quality Index (PSQI) [22]. The PSQI is a 19‐item questionnaire that evaluates sleep quality over the preceding month and generates a global score ranging from 0 to 21, with higher scores indicating poorer sleep quality. A global PSQI score ≥ 5 was considered indicative of clinically relevant poor sleep quality.

In addition to the global score, the following PSQI‐derived variables were examined to characterise specific aspects of sleep: total sleep duration, reported in hours and minutes; sleep latency, defined as the usual time (in minutes) required to fall asleep; sleep efficiency, calculated as the ratio of total sleep time to time spent in bed and expressed as a percentage.

#### 2.3.2 Insomnia Symptoms

Insomnia symptoms were assessed using the Insomnia Severity Index (ISI) [23] The ISI is a 7‐item questionnaire assessing perceived severity of difficulties with sleep initiation, sleep maintenance, early morning awakening, sleep dissatisfaction, interference with daytime functioning, noticeability of sleep problems, and distress caused by sleep difficulties over the preceding two weeks. Total ISI scores range from 0 to 28, with higher scores reflecting greater insomnia severity. An ISI score > 8 was used to indicate the presence of clinically relevant insomnia symptoms.

#### 2.3.3 Daytime Sleepiness

Daytime sleepiness was evaluated using the Epworth Sleepiness Scale (ESS) [24]. The ESS consists of 8 items assessing the likelihood of dozing in common daily situations, yielding a total score ranging from 0 to 24. Higher scores indicate greater daytime sleepiness, and a score ≥ 10 was considered indicative of excessive daytime sleepiness.

#### 2.3.4 Screening for Sleep‐Disordered Breathing Risk

Risk for sleep‐disordered breathing was screened using the STOP‐BANG questionnaire, an 8‐item instrument assessing snoring, tiredness, observed apneas, hypertension, body mass index, age, neck circumference and sex [25]. Higher STOP‐BANG scores indicate greater risk for sleep‐disordered breathing and were used as proxy measures for sleep phenotype classification in the absence of polysomnography.

### 2.4 Objective Nocturnal Respiratory Assessment

Objective nocturnal respiratory and oxygenation parameters were assessed using overnight pulse oximetry performed in the participant's habitual sleep environment. Pulse oximetry was used to quantify nocturnal oxygen desaturation and respiratory‐related oxygenation abnormalities in a pragmatic clinical setting [26].

#### 2.4.1 Oxygen Saturation Measures

Minimum oxygen saturation (Sat min), defined as the lowest oxygen saturation value recorded during the monitoring period, and maximum oxygen saturation (Sat max), defined as the highest oxygen saturation value recorded during the monitoring period.

#### 2.4.2 Oxygen Desaturation Index (ODI)

The ODI was calculated as the number of oxygen desaturation events per hour of recording, with desaturation events defined as a ≥ 3% reduction in oxygen saturation from baseline. ODI was used as an indicator of the frequency of nocturnal oxygen desaturation events.

#### 2.4.3 Time Spent in Hypoxemia

The percentage of total recording time spent with oxygen saturation below 90% (SpO_2_ < 90%) was calculated as a time‐based measure of nocturnal hypoxemia. This variable reflects the cumulative duration of reduced oxygen saturation during sleep and provides complementary information to event‐based indices such as ODI.

### 2.5 Operational Definitions of Sleep Groups

Sleep groups were defined a priori using combined subjective and objective criteria: Insomnia was defined by the presence of clinically relevant insomnia symptoms together with an ISI score > 8 and a PSQI score ≥ 5. Sleep‐disordered breathing (SDB) was defined by high‐risk STOP‐BANG screening, ESS ≥ 10, and ODI ≥ 15 events/h [27]. Comorbid insomnia and sleep‐disordered breathing (COMISA) was defined as the coexistence of both insomnia and SDB [27]. Participants meeting criteria for SDB without insomnia and those without sleep complaints were included for descriptive purposes but excluded from inferential analyses due to small sample sizes.

### 2.6 Statistical Analysis

All statistical analyses were performed using IBM SPSS Statistics (version 31). Continuous variables are presented as mean ± standard deviation (SD), and categorical variables are reported as frequencies and percentages. Normality of continuous variables was assessed using the Shapiro–Wilk test, and homogeneity of variances was evaluated using Levene's test. As this was a clinic‐based observational study with a fixed sample, no a priori sample size or power calculation was performed; results were therefore interpreted with emphasis on effect estimates and confidence intervals.

For our first objective, between‐group comparisons (insomnia vs. COMISA) were conducted using independent‐samples *t*‐tests for approximately normally distributed variables or Mann–Whitney U tests when distributional assumptions were not met. Group differences in categorical variables were assessed using chi‐square tests or Fisher's exact tests, as appropriate.

To address the second objective—to determine whether subjective sleep quality was associated with pain intensity across groups—we used the PSQI global score as the primary subjective sleep predictor because it captures overall perceived sleep quality across multiple domains and varies meaningfully within insomnia populations, rather than instruments such as the ISI, which primarily reflects insomnia symptom severity and was a defining characteristic of the study sample. Pain intensity was specified as the primary outcome. Thus, associations between PSQI global score and pain intensity were examined using univariate linear regression models, with sleep variables treated as continuous predictors. Given the a priori hypothesis of heterogeneity in sleep–pain relationships, all analyses were conducted separately within the insomnia and COMISA groups to allow group‐specific estimation of effect direction and magnitude. As sensitivity analyses, additional models were fitted adjusting for age, sex, BMI and ethnicity (coded as white vs. non‐white), both individually and simultaneously, to assess the robustness of observed associations to potential confounding. These covariates were selected a priori based on clinical relevance and known associations with sleep‐disordered breathing and pain [28, 29, 30], while primary analyses were conducted without adjustment to avoid overfitting and potential overadjustment given the modest sample size. In addition, supplementary interaction models including group, PSQI total score, and their interaction term were used to evaluate if the slope of the association between PSQI and pain intensity differed by group. To further refine the interpretation of global sleep–pain associations, additional exploratory analyses examined PSQI‐derived sleep parameters, including sleep duration, sleep latency and sleep efficiency, as predictors of pain intensity within each group.

Finally, for our third exploratory objective, i.e., to evaluate whether objective indices of nocturnal hypoxemia and autonomic activity are independently associated with pain intensity and other pain parameters, additional univariate linear regression analyses were conducted within each group. Objective predictors derived from overnight oximetry included minimum oxygen saturation, oxygen desaturation index, percentage of recording time with SpO_2_ < 90%, mean nocturnal heart rate, and maximum nocturnal heart rate. Pain intensity (VAS) served as the outcome variable in these exploratory models. All statistical tests were two‐tailed, and a *p*‐value < 0.05 was considered statistically significant.

Given the exploratory nature of the study and the modest sample size, results were interpreted with emphasis on effect direction and consistency rather than statistical significance alone, and no formal correction for multiple comparisons was applied.

## 3 Results

### 3.1 Sample Demographic and Pain Characteristics

The analytic sample comprised 50 patients with chronic TMD‐related orofacial pain, including 20 with insomnia alone (mean age 42.0 ± 10.4 years; 85.0% female) and 30 with COMISA (mean age 48.8 ± 13.1 years; 80.0% female). Between‐group comparisons of demographic characteristics, pain features, subjective sleep measures, and objective nocturnal respiratory and autonomic parameters are summarised in Tables 1 and 2.

**TABLE 1:** Demographic and pain characteristics of patients with insomnia and comorbid insomnia and sleep‐disordered breathing (COMISA).

Variable	Insomnia (*n* = 20)	COMISA (*n* = 30)	*p*
**Demographics**			
Age, years (mean ± SD)	42.0 ± 10.4	48.8 ± 13.1	0.12
Female gender, *n* (%)	17 (85.0)	24 (80.0)	0.72
Body mass index, kg/m^2^ (mean ± SD)	23.9 ± 3.3	27.7 ± 4.8	**0.01**
Diabetes, *n* (%)	1 (5.0)	0 (0.0)	0.40
Hypertension, *n* (%)	2 (10.0)	10 (33.3)	0.06
**Pain characteristics**			
Pain duration, years (mean ± SD)	5.5 ± 3.7	7.0 ± 5.2	0.42
Pain intensity (VAS 0–10), mean ± SD	7.45 ± 1.67	7.33 ± 1.90	0.95
Pain frequency, days/week (mean ± SD)	2.55 ± 1.19	3.37 ± 1.35	**0.04**
Headache present, *n* (%)	16 (80.0)	28 (93.3)	0.20
Myalgia present, *n* (%)	14 (70.0)	18 (60.0)	0.56
Myofascial pain present, *n* (%)	15 (75.0)	24 (80.0)	0.74
Temporalis muscle involvement, *n* (%)	14 (70.0)	26 (86.7)	0.17
Masseter muscle involvement, *n* (%)	14 (70.0)	26 (86.7)	0.15
TMJ involvement, *n* (%)	14 (70.0)	8 (26.7)	**0.002**
Cervical muscle involvement, *n* (%)	5 (25.0)	7 (23.3)	0.89
SCM involvement, *n* (%)	1 (5.0)	1 (3.3)	1.00
Occipital region involvement, *n* (%)	3 (15.0)	9 (30.0)	0.22
**Circadian pain pattern**			
Morning pain present, *n* (%)	10 (50.0)	28 (93.3)	< **0.001**
Afternoon pain present, *n* (%)	16 (80.0)	27 (90.0)	0.42
Night pain present, *n* (%)	0 (0.0)	7 (23.3)	**0.02**

*Note:* Bold values indicate statistical significance set as *p* < 0.05.

Abbreviations: COMISA, comorbid insomnia and sleep‐disordered breathing; kg/m^2^, kilograms per square metre; *n*, number; SCM, sternocleidomastoid muscle; SD, standard deviation; TMJ, temporomandibular joint; VAS, Visual Analog Scale.

**TABLE 2:** Subjective sleep and respiratory measures in patients with insomnia and comorbid insomnia and sleep‐disordered breathing (COMISA).

Variable	Insomnia (*n* = 20)	COMISA (*n* = 30)	*p*
**Subjective sleep measures**			
ISI total score (mean ± SD)	16.8 ± 4.6	15.4 ± 4.1	0.27
PSQI total score (mean ± SD)	13.45 ± 3.44	13.00 ± 3.96	0.76
Epworth Sleepiness Scale (mean ± SD)	9.2 ± 4.7	8.7 ± 4.4	0.54
High STOP‐BANG risk, *n* (%)	5 (25.0)	26 (86.7)	**< 0.001**
Sleep duration, hours (mean ± SD)	8.80 ± 0.92	8.38 ± 1.70	0.25
Sleep duration, minutes (mean ± SD)	528 ± 55	503 ± 102	0.25
Sleep latency, minutes (mean ± SD)	60.5 ± 56.5	47.5 ± 42.4	0.60
Sleep efficiency, % (mean ± SD)	65.6 ± 15.3	69.2 ± 15.2	0.31
**Nocturnal respiratory and autonomic measures**			
Minimum oxygen saturation, % (mean ± SD)	91.9 ± 2.4	80.4 ± 8.4	**< 0.001**
Maximum oxygen saturation, % (mean ± SD)	97.6 ± 1.2	95.1 ± 1.9	**< 0.001**
Time with SpO_2_ < 90%, % (mean ± SD)	0.76 ± 2.94	7.22 ± 13.82	**< 0.001**
Oxygen desaturation index (ODI)	1.80 ± 1.40	25.26 ± 20.59	**< 0.001**
Mean heart rate, bpm (mean ± SD)	58.5 ± 6.3	61.2 ± 8.3	0.30
Maximum heart rate, bpm (mean ± SD)	93.4 ± 10.2	92.0 ± 12.4	0.68

*Note:* Bold values indicate statistical significance set as *p* < 0.05.

Abbreviations: bpm, beats per minute; COMISA, comorbid insomnia and sleep‐disordered breathing; ISI, Insomnia Severity Index; ODI, Oxygen Desaturation Index; PSQI, Pittsburgh Sleep Quality Index; SD, standard deviation; SpO_2_, peripheral oxygen saturation; STOP‐BANG, Snoring, Tiredness, Observed apnea, high blood Pressure, Body mass index, Age, Neck circumference, Gender.

Patients with COMISA had a significantly higher body mass index than those with insomnia alone (27.7 ± 4.8 vs. 23.9 ± 3.3 kg/m^2^; *p* = 0.01). Age and sex distribution did not differ significantly between groups. Hypertension was more prevalent in the COMISA group, showing a trend towards significance (*p* = 0.06), whereas diabetes prevalence was low and comparable between groups.

Regarding pain characteristics, pain intensity did not differ between insomnia and COMISA groups (VAS: 7.45 ± 1.67 vs. 7.33 ± 1.90; *p* = 0.95), nor did pain duration. Pain frequency (days/week) was higher in COMISA (*p* = 0.04) and is reported descriptively but was not retained as an analytic outcome.

Marked between‐group differences were observed in circadian pain patterns. Morning pain was substantially more prevalent in COMISA than insomnia (93.3% vs. 50.0%; *p* < 0.001), and night pain was reported exclusively in the COMISA group (23.3% vs. 0%; *p* = 0.02). Afternoon pain prevalence did not differ between groups.

In terms of pain distribution, temporomandibular joint involvement was significantly more frequent in the insomnia group than in COMISA (70.0% vs. 26.7%; *p* = 0.002). No significant group differences were observed for temporalis, masseter, cervical, sternocleidomastoid, or occipital region involvement. Headache, myalgia and myofascial pain were common in both groups, with no statistically significant differences.

### 3.2 Subjective Sleep Characteristics

Despite differing physiological sleep profiles, subjective sleep complaints were broadly comparable between groups (Table 2). Insomnia Severity Index, PSQI global score, Epworth Sleepiness Scale score, sleep duration, sleep latency and sleep efficiency did not differ significantly between insomnia and COMISA patients (all *p* > 0.25).

In contrast, a markedly higher proportion of COMISA patients screened positive for high STOP‐BANG risk compared with those with insomnia alone (86.7% vs. 25.0%; *p* < 0.001), consistent with the presence of sleep‐disordered breathing in this group.

### 3.3 Objective Nocturnal Respiratory and Autonomic Measures

Objective nocturnal parameters derived from overnight oximetry revealed pronounced physiological differences between groups (Table 2). Compared with insomnia, COMISA patients exhibited significantly lower minimum oxygen saturation (80.4% ± 8.4% vs. 91.9% ± 2.4%; *p* < 0.001), lower maximum oxygen saturation (95.1% ± 1.9% vs. 97.6% ± 1.2%; *p* < 0.001), greater percentage of recording time with SpO_2_ < 90% (7.22% ± 13.82% vs. 0.76% ± 2.94%; *p* < 0.001), and a markedly higher oxygen desaturation index (25.26 ± 20.59 vs. 1.80 ± 1.40 events/h; *p* < 0.001). Mean and maximum nocturnal heart rate did not differ significantly between groups.

### 3.4 Associations Between Subjective Sleep Quality and Pain Intensity

Linear regression analyses were conducted separately within each group to evaluate whether global subjective sleep quality, assessed using the PSQI total score, was associated with pain intensity (VAS).

**FIGURE 1:**
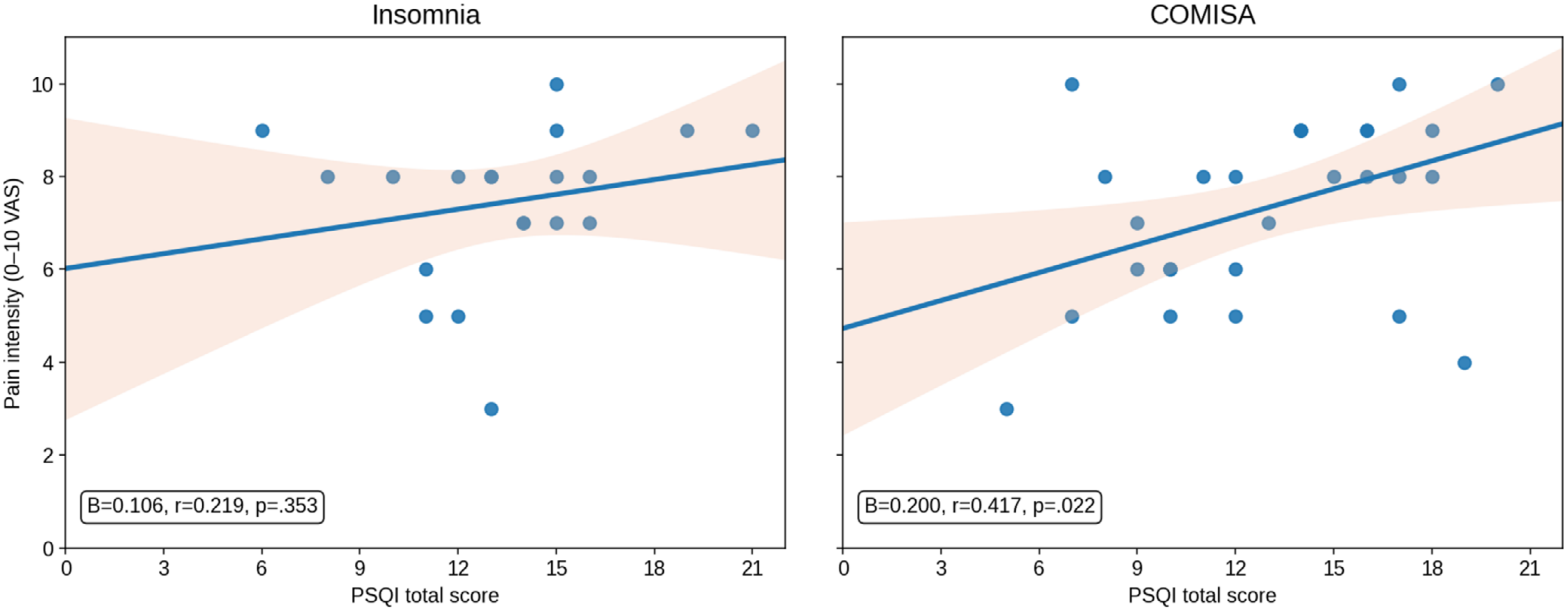
Unadjusted associations between subjective sleep quality and pain intensity by group. Each point represents an individual participant. Because pain intensity and PSQI scores are discrete variables, multiple observations share identical values and may therefore overlap. Lines represent unadjusted linear regression fits within each group; shaded areas indicate 95% confidence intervals.

In the insomnia group, PSQI total score was not significantly associated with pain intensity (*B* = 0.106, *r* = 0.219, *p* = 0.353; 95% CI: −0.128 to 0.341), indicating no meaningful relationship between global subjective sleep quality and pain severity (Figure 1).

In contrast, in the COMISA group, PSQI total score was positively and significantly associated with pain intensity (*B* = 0.200, *r* = 0.417, *p* = 0.022), accounting for approximately 17% of the variance in pain intensity (Figure 1). Higher levels of subjective sleep disturbance were associated with greater pain severity in this group.

Formal interaction models including group, PSQI total score, and their interaction term did not reach statistical significance (*B* = 0.200, *r* = 0.417, *p* = 0.022; 95% CI: 0.032–0.369), likely reflecting limited statistical power; however, the stratified analyses demonstrated a clear divergence in sleep–pain associations between insomnia and COMISA groups.

After adjustment for BMI, age, gender and ethnicity, PSQI total score remained significantly associated with pain intensity (*B* = 0.175, *p* = 0.040; 95% CI: 0.008–0.342). The adjusted model was significant overall (*R* = 0.620, *R* ^2^ = 0.384; adjusted *R* ^2^ = 0.256; *F*(5, 24) = 2.995, *p* = 0.031), while none of the covariates were individually significant (BMI *p* = 0.662; age *p* = 0.651; gender *p* = 0.070; ethnicity *p* = 0.164).

### 3.5 Associations Between Specific Sleep Parameters and Pain Intensity

Exploratory regression analyses examined PSQI‐derived sleep efficiency, total sleep duration, and sleep latency as predictors of pain intensity within each group in univariate models.

Neither insomnia nor COMISA groups showed significant associations between pain intensity and sleep efficiency, total sleep duration, or sleep latency (all *p* > 0.20), suggesting that global subjective sleep disturbance rather than specific sleep timing or efficiency parameters was most relevant to pain severity in COMISA.

### 3.6 Objective Nocturnal Physiology and Pain Intensity

To determine whether objective nocturnal physiological parameters were associated with pain outcomes, separate linear regression analyses were conducted within each group with pain intensity.

In the insomnia group, none of the objective nocturnal respiratory or autonomic measures were significantly associated with pain intensity. Specifically, minimum oxygen saturation, oxygen desaturation index, percentage of recording time spent with oxygen saturation below 90%, mean nocturnal heart rate, and maximum nocturnal heart rate all showed non‐significant relationships with pain severity (all *p* ≥ 0.10).

In contrast, within the COMISA group, selected objective physiological indices were significantly associated with pain intensity. Lower minimum oxygen saturation was associated with greater pain intensity (*β* = 0.371, *p* = 0.043; 95% CI: 0.003 to 0.166), accounting for approximately 14% of the variance in pain severity. In addition, maximum nocturnal heart rate was inversely associated with pain intensity (*β* = −0.420, *p* = 0.021; −0.119 to −0.011), explaining approximately 18% of the variance.

## 4 Discussion

To our knowledge, this is the first study to examine sleep–pain relationships in chronic TMD pain in the context of COMISA, by explicitly distinguishing insomnia with or in isolation from COMISA. By integrating subjective sleep measures with objective nocturnal respiratory and autonomic indices, our findings demonstrate that sleep–pain associations in TMD are not uniform. Although pain intensity did not differ between groups, patients with COMISA experienced more frequent pain and distinct circadian pain patterns compared with those with insomnia alone. Importantly, subjective sleep disturbance was associated with pain intensity in COMISA, whereas in insomnia alone, subjective sleep quality was not related to pain intensity. Furthermore, objective nocturnal physiological markers were associated with pain intensity only in COMISA, with lower minimum oxygen saturation and altered autonomic activity showing significant relationships with pain intensity, suggesting that sleep disturbances in insomnia alone, characterised primarily by subjective sleep quality, and sleep disturbance in COMISA, characterised by both subjective complaints and nocturnal physiological stress, represent qualitatively different sleep‐related exposures in TMD, with distinct implications for pain intensity.

Both samples did not differ in terms of age, gender distribution, pain intensity, or pain duration. However, it is important to note that pain intensity was moderate to high, which may differ from other TMD clinical samples [31, 32, 33]. As expected, BMI was significantly higher in the COMISA group, whereas pain frequency (in number of days per week) was also slightly higher in this group. In terms of pain location and distribution, everything remained similar between groups except a major involvement of the TMJ in the insomnia group. However, an interesting finding emerged in temporal patterns of pain, as COMISA patients reported the presence of pain more commonly in the morning (almost all participants) and slightly more at night than their insomnia counterparts. Morning pain, and headache in particular, has been frequently reported in association with sleep‐disordered breathing, including obstructive sleep apnea [34]. Proposed mechanisms include nocturnal hypoxemia and respiratory‐related sleep fragmentation, which may be most evident upon awakening. In this context, the higher prevalence of morning pain in the COMISA group may be consistent with the lower minimum oxygen saturation, higher oxygen desaturation index, and greater percentage of recording time with SpO_2_ < 90% observed in this group, although causal inferences cannot be made.

Regarding subjective sleep measures, consistent with other studies in chronic pain, both groups did not significantly differ [35]. However, the absence of an association between sleep quality and pain intensity in patients with insomnia alone may appear inconsistent with prior literature linking poor sleep to heightened pain [36, 37]. This finding should be interpreted in light of the chronicity and severity of pain in the present sample, the clinical setting, and the sleep phenotype under study. Participants were recruited from a specialised orofacial pain clinic and reported uniformly high levels of pain intensity, which may have limited the dynamic range available to detect additional sleep‐related modulation of pain severity, raising the possibility of a ceiling effect. Much of the literature demonstrating poor sleep‐related increases in pain intensity is derived from more heterogeneous clinical samples in which sleep‐disordered breathing is not systematically assessed; observed sleep–pain associations may partly reflect unrecognised physiological sleep disturbances rather than insomnia symptoms alone. Moreover, the cross‐sectional design of the present study captures a single time point in patients with longstanding TMD‐related pain, precluding inferences about temporal ordering or dynamic amplification processes. Finally, as noted in both the standard deviations and the visual distribution in Figure 1, a slight greater variability in pain intensity and subjective sleep quality among patients with COMISA compared with those with insomnia alone was observed. This broader range of both sleep disturbance and pain severity in the COMISA group may have increased the ability to detect sleep–pain associations in that phenotype, consistent with prior reports highlighting substantial heterogeneity in pain and sleep severity among individuals with comorbid insomnia and sleep apnea [35, 38]. Differences in group size may have contributed to the observed patterns; nevertheless, the consistency in the direction of associations across subjective and objective measures suggests that variability in clinical expression, rather than sample size alone, may play an important role. This is in line with prior studies proposing that COMISA represents a distinct clinical phenotype with pain correlates that differ from those observed in insomnia or sleep‐disordered breathing alone [35, 38]. The limited sample size (*n* = 20), combined with the restricted variability in both PSQI scores and pain intensity, substantially reduces the ability to detect small‐to‐moderate effects. Thus, the null findings in this group may reflect limited statistical sensitivity rather than a true absence of association.

In the COMISA group, however, a different and more expected pattern emerged, with global subjective sleep quality being significantly associated with pain intensity. Furthermore, although no specific subjective sleep parameter (e.g., sleep efficiency, duration, or latency) appeared to predominantly drive this association, lower minimum oxygen saturation and altered nocturnal autonomic activity, indexed by maximum heart rate, were significantly associated with pain intensity. These findings are consistent with recent PSG‐based analyses showing that COMISA is characterised by distinct patterns of nocturnal autonomic dysregulation, including reduced parasympathetic activity during wakefulness and increased sympathetic activation during sleep [39]. For instance, it has been suggested that COMISA‐related hyperarousal, arousal instability, and subtle behavioural markers such as nocturnal yawning that may reflect latent insomnia physiology within SDB presentations [40]. Although speculative, our findings also raise the possibility that pain severity in COMISA is influenced more by peak nocturnal physiological stressors than by cumulative hypoxemic burden or average autonomic tone. This interpretation is supported by prior polysomnography‐based studies showing that oxygen saturation nadir, but not cumulative indices such as apnea–hypopnea index or total hypoxemia time, was associated with pain outcomes [41]. Hypothetically, the association with lower oxygen nadir suggests that the severity of the most pronounced hypoxemic events may be more relevant to pain intensity than the overall frequency or duration of desaturation, while the inverse association with maximum heart rate points to altered or constrained autonomic reactivity rather than generalised hyperarousal in this sample. However, these interpretations should be considered as tentative and should be approached cautiously, as oximetry‐based SDB classification offers less physiological detail and contextualization than PSG‐derived measures.

To our knowledge, this is the first study to specifically investigate COMISA in a TMD population and to examine its relationship with pain outcomes, providing valuable information regarding this emerging condition. However, several limitations should be considered. First, although the study design aimed to include an obstructive sleep apnea–only group (i.e., sleep‐disordered breathing without clinically relevant insomnia), only two individuals met criteria for sleep‐disordered breathing in the absence of insomnia symptoms, precluding meaningful statistical analysis. Accordingly, the absence of a sleep‐disordered breathing–only group substantially restricts the interpretability of COMISA‐specific conclusions, as it prevents isolating the independent effects of SDB from the combined COMISA phenotype. This limitation is consistent with prior clinic‐based orofacial pain cohorts, in which isolated SDB appears substantially less prevalent than insomnia or COMISA presentation [27]. As a result, the present findings cannot disentangle the independent contribution of sleep‐disordered breathing alone from that of COMISA, and comparisons are limited to insomnia alone versus COMISA. Future studies recruiting from sleep clinics or community‐based samples will be needed to directly compare insomnia, sleep‐disordered breathing alone, and COMISA in TMD.

Second, the cross‐sectional design limits causal inference regarding the directionality of sleep–pain relationships. While our analyses identify distinct patterns of association between sleep variables and pain outcomes across groups, longitudinal or experimental designs will be required to determine whether sleep disturbance contributes to pain progression, whether pain disrupts sleep, or whether both are driven by shared mechanisms.

Third, the classification of individuals with sleep‐disordered breathing was based on questionnaires such as STOP‐BANG, ESS and oximetry‐derived ODI rather than the apnea–hypopnea index obtained from polysomnography. This approach, while clinically pragmatic and used in different contexts [42], introduces a risk of misclassification of SDB severity and type. Although oximetry provides clinically meaningful indices of nocturnal hypoxemia and respiratory disturbance and has shown to be valid vs. PSG home testing [43], it does not capture sleep architecture, arousal indices, or microstructural sleep features that may also influence pain processing. Accordingly, in the present study COMISA classification and physiological interpretations should be viewed as oximetry‐based proxies rather than full PSG‐derived characterizations.

Finally, the modest sample size constitutes a major limitation, possibly increasing the risk of type II error, and may yield less stable regression estimates—particularly for subgroup‐specific models and interaction testing. Accordingly, the absence of statistically significant interactions should not be interpreted as evidence of no differential association, and subgroup‐specific findings should be considered preliminary and hypothesis‐generating pending replication in larger samples using polysomnography.

## 5 Conclusion

Overall, the present findings highlight the potential relevance of COMISA in shaping sleep–pain relationships in chronic TMD‐related orofacial pain. By showing that associations between sleep quality, nocturnal physiological measures, and pain intensity were more evident in COMISA than in insomnia alone, this study underlines the need to consider COMISA as a distinct and understudied sleep phenotype in pain research. Although the cross‐sectional design precludes causal inference, these results support the importance of further, more detailed investigation of COMISA in relation to pain, using large multicenter samples, longitudinal designs, and comprehensive sleep assessments (e.g., polysomnography).

## Funding

The authors have nothing to report.

## Conflicts of Interest

The authors declare no conflicts of interest.

## Data Availability

The data that support the findings of this study are available from the corresponding author upon reasonable request.
